# Demographic and clinical profile of patients treated with proximal femoral nails – a 10-year analysis of more than 40,000 Cases

**DOI:** 10.1186/s12891-022-05772-1

**Published:** 2022-09-01

**Authors:** Christopher G. Finkemeier, Chantal E. Holy, Jill W. Ruppenkamp, Mollie Vanderkarr, C. Sparks

**Affiliations:** 1 Sutter Health, Carmichael, CA USA; 2 MedTech Epidemiology, Johnson & Johnson, New Brunswick, NJ USA; 3 DePuy Synthes, West Chester, PA USA

**Keywords:** Fracture fixation, Intramedullary, Retrospective studies, Length of stay, Patient discharge, Reoperation

## Abstract

**Background:**

Hip fractures are common in elderly populations and can be life threatening. Changes in healthcare delivery and outcomes for patients with hip fracture treated with intramedullary nails are not well characterized. The objectives of our study were: 1) the characterization of patients treated with the Trochanteric Fixation Nail -Advanced™(TFNA) Proximal Femoral Nailing System or comparable nails (index) and estimate 12-month all-cause readmissions (ACR) and reoperations following index; and 2) the evaluation of 10-year healthcare utilization (HCU) trends for treatment of femoral fractures with femoral nails.

**Methods:**

This is a retrospective database analysis using the Premier hospital database. All adults with femoral fracture treated with an intramedullary nail, from 2010 to Q3 2019, in the inpatient setting, were identified. Exclusion criteria included patients with bilateral hip surgery and presence of breakage at time of initial surgery. The primary outcome was ACR and reoperation, the secondary outcomes were healthcare utilization metrics. Variables included demographics, comorbidities (Elixhauser Index (EI)), surgical intervention variables and hospital characteristics.

**Results:**

Forty-one thousand one hundred four patients were included in the study, of which 14,069 TFNA patients, with average age 77.9 (Standard deviation (SD): 12.0), more than 60% with 3 or more comorbidities (more than 64% for TFNA), 40% with severe or extreme disease severity and one third with severe or extreme risk for mortality. ACR reached 60.1% (95% confidence interval (CI): 59.6%-60.5%) – for TFNA: 60.0% (95%CI: 59.2%-60.8%). The reoperation rate was 4.0% (95%CI: 3.8%-4.2%) – for TFNA: 3.8% (95%CI: 3.5%-4.1%). Length of stay (LOS) averaged 5.8 days (SD: 4.8), and 12-month hip reoperation was 4.0% (3.8%-4.2%), in TFNA cohort: 3.8% (3.5%-4.1%). From 2010 to 2019: the percentage patients operated within 48 h of admission significantly increased, from 75.2% (95%CI: 74.3%-76.1%) to 84.3% (95%CI: 83.9%-84.6%); LOS significantly decreased, from 6.2 (95%CI: 6.0–6.4) to 5.6 (95%CI: 5.5–5.7) days; discharge to skilled nursing facilities (SNF) increased from 56.0% (95%CI: 54.8%-57.2%) to 61.5% (95%CI: 60.8%-62.2%); ACR rates decreased but reoperation rates remained constant.

**Conclusions:**

ACR and reoperation rates were similar across device types and averaged 60.1% and 4.0%, respectively. Ten-year analyses showed reductions in hospital HCU and greater reliance on SNF.

## Background

Femoral fractures are one of the most common fall injuries in older adults. A total of 325,420 elderly patients were hospitalized for femoral fractures in 2018, of which 67% were female. This higher rate among the female population may be due to underlying osteoporosis [1]. Common surgical treatment of proximal femoral fractures includes the use of intramedullary nails. Intramedullary nails are long implants that allow soft-tissue preservation, load sharing and overall low risk of wound complications due to implantation within the medullary cavity [2–5].

There are many different nail systems available to surgeons, but no difference in outcomes between nails has been found in a 2014 Cochrane review and a recent meta-analysis [6, 7]. Most studies, however are small and of relative low generalizability [7].

In 2015 a novel nail system was brought to market: the DePuy Synthes Trochanteric Fixation Nail Advanced™(TFNA) Proximal Femoral Nailing System. This device is different from other marketed nails as it was designed with a smaller proximal diameter – to allow greater preservation of native bone—but also a far stronger material, to reduce risk of breakage. As expected, a recent study looking at rate of nail breakage from TFNA vs that of other devices revealed no difference, and a 7,979 patients study from the Kaiser healthcare system further confirmed this finding [8, 9].

Wallace et al. used a large hospital billing database to evaluate nail fracture rates in patients treated with TFNA vs similar devices (Stryker® Gamma3® and Zimmer® Natural Nail®), and created a large dataset of nearly 40 K patients, of which 14,370 were implanted with TFNA.

Beyond nail fracture, we were interested in characterizing the demographic and comorbid conditions of patients treated with these nails and estimate the all-cause readmissions and hip-related reoperations across all 40,000 patients. For this study, we used the population identified by Wallace et al.and expanded the analyses to include additional outcomes, specifically all-cause readmissions and hip-related readmissions and reoperations.

## Methods

### Data

This is a retrospective study using the patient cohort defined by Wallace et al. [8] from the Premier Healthcare Database (PHD). This database stems from a health care alliance, which was formed for hospitals to share knowledge, improve patient safety, and reduce risks. The PHD contains complete clinical coding, including diagnosis, procedures, and hospital-prescribed medications from more than 20% of all hospital admissions throughout the United States (> 1040 hospitals and hospital systems). The PHD collects data from participating hospitals in its health care alliance. Participation in the PHD health care alliance is voluntary. Although the database excludes federally funded hospitals (e.g., Veterans Affairs), the hospitals included are nationally representative based on bed size, geographic region, location (urban/rural) and teaching hospital status. The database contains a date-stamped log of all billed items by cost-accounting department including medications; laboratory, diagnostic, and therapeutic services; and primary and secondary diagnoses for each patient’s hospitalization. Identifier-linked enrollment files provide demographic and payor information. Detailed service level information for each hospital day is recorded; this includes details on medication and devices received.

### Institutional Review Board (IRB)

The study used PHD de-identified that cannot be re-identified. As such, the use of the PHD is exempt from broad IRB approval.

### Cohort

Patients were identified as being > 21 years of age, having an International Classification of Disease (ICD) procedure code specific for femur fracture repair and being treated with TFNA. For contextualization, patients treated with a nail similar in design to TFNA were included and categorized as “non-TFNA nail” patients. These included the Titanium Trochanteric Fixation Nail (TFN – DePuy Synthes, PA – USA), Gamma3® (Stryker®, MI – USA) or Natural Nail® (Zimmer®, IN – USA), between January 1^st^, 2010, to September 30^th^, 2019. The end date was selected to pre-date the COVID-19 pandemic and its impact on patients and hospitals. Exclusion criteria included missingness (missing sex or age), presence of nail breakage concurrent with the first femoral fracture repair surgery, and presence of bilateral fractures, as these patients may be at greater risk for surgical complications.

### Intervention

All patients underwent fracture repair using one of the nails mentioned above. The date of the intervention is defined as the “index”.

### Variables

The following variables were collected at time of index for all patients: demographics (age, gender, race), 31 chronic comorbidities as defined by the Elixhauser Comorbidity Index, [10] presence of delirium, dementia, osteoporosis, atrial fibrillation, coma, concurrent fractures (rib fracture, pelvic fractures, foot and other long bone fractures, head injuries), Abbreviated Injury Scales (AIS) for each anatomy, Injury Severity Score (ISS). The following variables were collected at any time during the admission: vitals, medications (blood thinners, metformin, insulin, anti-inflammatories, narcotics, osteoporosis drugs), laboratory values (HbA1c, bone mineral density – when available, blood panel values). The following variables were collected after surgery: blood unit used, implant construct (long vs short). The following variables were collected specifically to characterize the fracture type: open vs closed fracture, fracture type (subtrochanteric, pertrochanteric, intertrochanteric, femoral neck, femoral shaft, reverse oblique fracture, pathological fractures, bone neoplasm.) Variables specific to the hospital included: hospital size (by number of beds), hospital teaching status and census location.

### Outcomes

The following outcomes were evaluated: operating room time (ORT) in minutes, length of hospital stay (LOS) in days, discharge destination (home vs home health vs SNF/other high acuity settings), 12-months reoperation rates and 12-months all-cause readmission rates. Reoperations were defined as any new hip procedure, whereas all-cause readmission was defined as any new inpatient admission.

### Analyses

Two types of analyses were conducted: 1) a descriptive analysis of all variables and outcomes, for each device type; and 2) a longitudinal analysis of patient comorbidity and HCU, over the 10-year period of the study.

For the descriptive analyses by device type, counts and proportions were provided for dichotomous and polychotomous variables. Measures of central tendency and spread were provided for continuous variables. All findings were expressed with mean and 95% confidence intervals. A generalized linear model (GLM) was built for each of the 4 distinct population (one for patients treated with TFNA, one for TFN, one for Gamma3, one for Natural Nail). The GLM model (family: gamma, link = “log”) was used to estimate adjusted length of stay per fracture type. (Gamma regression models, with positively skewed distributions, have been previously shown to be well suited for LOS analyses) [11–13]. Readmission and reoperation rates were estimated using a Poisson regression.

For longitudinal analyses over time, counts and proportions were provided for dichotomous (percentage operations within 48 h or admission, SNF discharge, readmission and reoperation rates) variables. Measures of central tendency and spread were provided for continuous variables (LOS, Elixhauser Index, ORT, age). All findings were expressed with mean and 95% confidence intervals.

All analyses were conducted using R (version 3.6.3).

## Results

### Population

A total of 41,104 patients were included in the study, 14,069 TFNA, 15,117 TFN, 1,776 Natural Nail and 10,142 Gamma3. Demographic and comorbid data for all patients are shown in Table 1.

**Table 1:** Age, gender, race, and comorbidity of patients included in the study, by device type

Variables	All Patients	TFNA-only	Other nails (non-TFNA) only
**N**	41,104	14,069	27,035
**Age (mean, (standard deviation))**	**77.9 (SD:12.0)**	**77.7 (SD:12.1)**	**78.0 (SD:11.9)**
**Age Category**			
less than 60	3,436 (8.4%)	1,163 (8.3%)	2,273 (8.4%)
60 to 69	5,299 (12.9%)	1,886 (13.4%)	3,413 (12.6%)
70 to 79	9,169 (22.3%)	3,262 (23.2%)	5,907 (21.8%)
80 to 84	7,101 (17.3%)	2,295 (16.3%)	4,806 (17.8%)
85 and above	16,099 (39.2%)	5,463 (38.8%)	10,636 (39.3%)
**Gender: Female**	**28,716 (69.9%)**	**9,691 (68.9%)**	**19,025 (70.4%)**
**Race**			
African American	1,630 (4.0%)	519 (3.7%)	1,111 (4.1%)
Caucasian	35,463 (86.3%)	12,561 (89.3%)	22,902 (84.7%)
Hispanic	79 (0.2%)	-	79 (0.3%)
NA	780 (1.9%)	278 (2.0%)	502 (1.9%)
Other	2,751 (6.7%)	499 (3.5%)	2,252 (8.3%)
Unavailable	401 (1.0%)	212 (1.5%)	189 (0.7%)
**Elixhauser Score (mean, (standard deviation))**	**3.3 (SD:2.0)**	**3.4 (SD:2.1)**	**3.2 (SD:2.0)**
**Elixhauser Category**			
0	2,141 (5.2%)	668 (4.7%)	1,473 (5.4%)
1–2	13,673 (33.3%)	4,406 (31.3%)	9,267 (34.3%)
3–4	15,074 (36.7%)	5,121 (36.4%)	9,953 (36.8%)
5 +	10,216 (24.9%)	3,874 (27.5%)	6,342 (23.5%)
Severity (1 to 4)*			
1	6,342 (15.4%)	2,265 (16.1%)	4,077 (15.1%)
2	18,340 (44.6%)	6,168 (43.8%)	12,172 (45.0%)
3	12,940 (31.5%)	4,398 (31.3%)	8,542 (31.6%)
4	3,482 (8.5%)	1,238 (8.8%)	2,244 (8.3%)
Risk of Mortality (1 to 4)*			
1	10,503 (25.6%)	3,517 (25.0%)	6,986 (25.8%)
2	16,552 (40.3%)	5,442 (38.7%)	11,110 (41.1%)
3	10,967 (26.7%)	4,003 (28.5%)	6,964 (25.8%)
4	3,082 (7.5%)	1,107 (7.9%)	1,975 (7.3%)
**Mortality during Index**	**810 (2.0%)**	**259 (1.8%)**	**551 (2.0%)**
**Femoral Shaft Fracture**	**966 (2.4%)**	**359 (2.6%)**	**607 (2.2%)**
**Head Injury**	**1,545 (3.8%)**	**996 (7.1%)**	**549 (2.0%)**
**Intensive Care Unit Use**	**3,835 (9.3%)**	**1,322 (9.4%)**	**2,513 (9.3%)**

^*^Risk of severity was based on the All Patient Refined (APR) hospital assessment of minor, moderate, severe or extreme, and is defined as the “extent of physiological decompensation or organ system function loss”. The APR mortality risk is simply defined as the “likelihood of dying”

The majority of patients were female (70%), Caucasian (86%), elderly (average age: 77.9), with nearly 40% age 85 and older. Comorbidities were frequent, with average Elixhauser index above 3 (suggesting more than 3 chronic comorbidity per patient) [10]. Nearly one quarter of all patients had 5 or more comorbidities. Using the hospital assessments of severity and mortality risks, 40% of all patients had a severe or extreme severity rating, and more than a third had a mortality risk of severe or extreme. Two percent of patients died during the index admission. Shaft fractures and head injury affected 2.5% and 3.8% of all patients. Intensive care units were used for more than 9% of all patients. The demographics and comorbidities were generally similar between patients treated with TFNA and the other nails.

### Operating room time, length of stay, discharge disposition, reoperations and readmissions

Average ORT was nearly 2 h (111.2 min, standard deviation (SD): 93.9), and LOS averaged 5.8 days (SD: 4.8). Only 14% were discharged to home, of which 7% with home health. Within 12 months of index, nearly 60% patients had a new hospital inpatient admission (ACR). Approximately 4% patients required a hip reoperation. The findings for the TFNA cohort and the cohort of patients treated with other comparable nails are detailed on Table 2.

**Table 2:** Operating room time, length of hospital stay, discharge disposition, and 12-month all-cause readmission and reoperation rates of patients treated with TFNA or other comparable implants

Outcomes	All Patients	TFNA only	Other Nails (non-TFNA) only
**Operating Room Time (minutes—mean (SD))**	**111.2 (SD: 93.9)**	**106.3 (SD: 46.8)**	**113.8 (SD: 111.5)**
**Length of stay (days—mean (SD))**	**5.8 (SD: 4.8)**	**5.7 (SD: 4.7)**	**5.9 (SD: 4.9)**
**Discharge**			
Home Health	2,993 (7.3%)	1,122 (8.0%)	1,871 (6.9%)
Home	2,750 (6.7%)	981 (7.0%)	1,769 (6.5%)
SNF & Other High-Acuity Settings	35,361 (86.0%)	11,966 (85.1%)	23,395 (86.5%)
12-Month Rate of Reoperation*	**4.0% (95%CI: 3.8%-4.2%)**	**3.8% (95%CI: 3.5%-4.1%)**	**4.1% (95%CI: 3.9%-4.4%)**
12-Month Rate of All-Cause Readmission*	**60.1% (95%CI: 59.6%-60.5%)**	**60.0% (95%CI: 59.2%-60.8%)**	**60.1% (59.5%-60.7%)**

For 12-month outcomes, only patients with complete 12-month data were included: All patients: 37,995; TFNA: 12,410 and Other Nails: 25,585

### Comorbidity and HCU trends over time

**Fig. 1:**
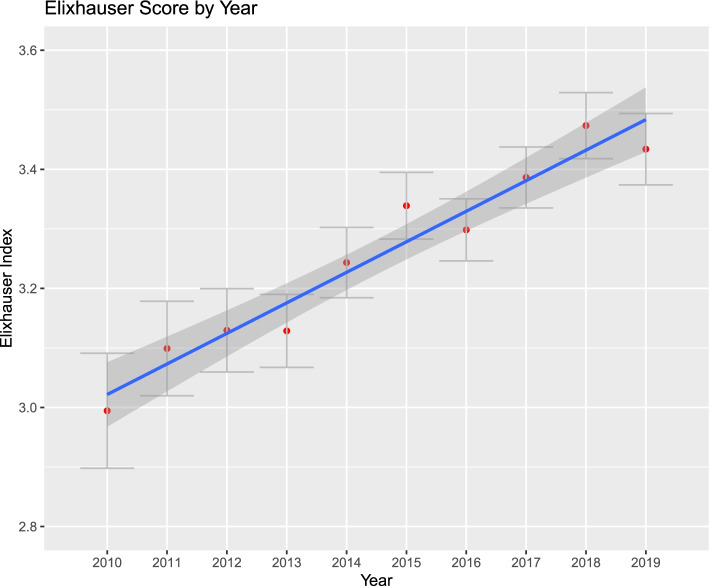
Average Elixhauser Index from 2010 to 2019. The Elixhauser Index (equivalent to a count of concurrent comorbidities) increased from 3.0 (95%CI: 2.9–3.1) to 3.4 (95%CI: 3.3–3.5)

**Fig. 2:**
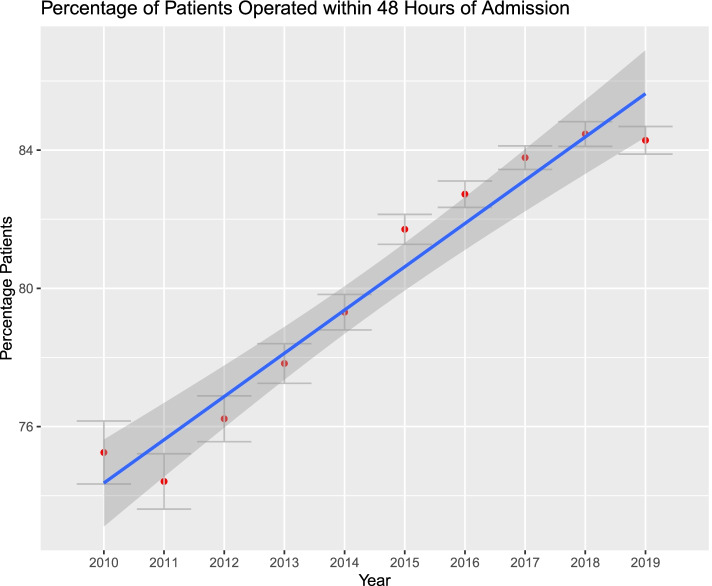
Percentage of patients surgically-treated within 48 h of admission, from 2010 to 2019. The percentage patients operated within that time frame increased from 75.2% (95%CI: 74.3%-76.1%) to 84.3% (95%CI: 83.9%-84.6%)

**Fig. 3:**
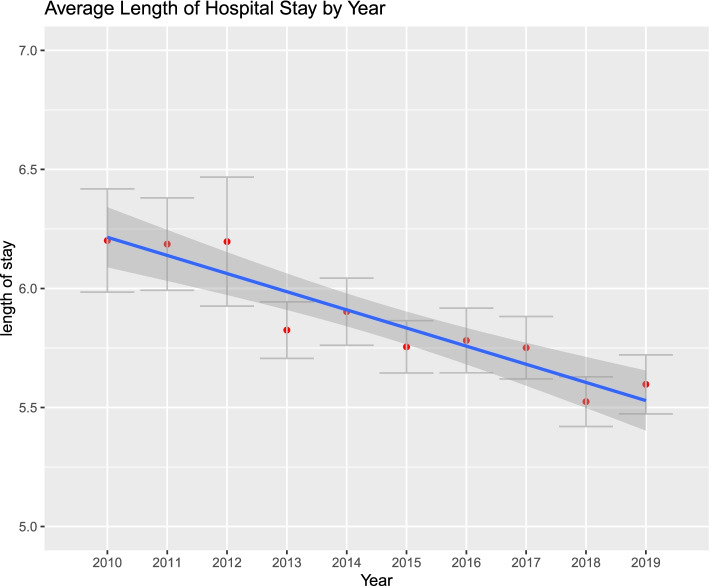
Average length of hospital stay from 2010 to 2019, in days. The length of hospital stay decreased from 6.2 (95%CI: 6.0–6.4) to 5.6 (95%CI: 5.5–5.7) days

**Fig. 4:**
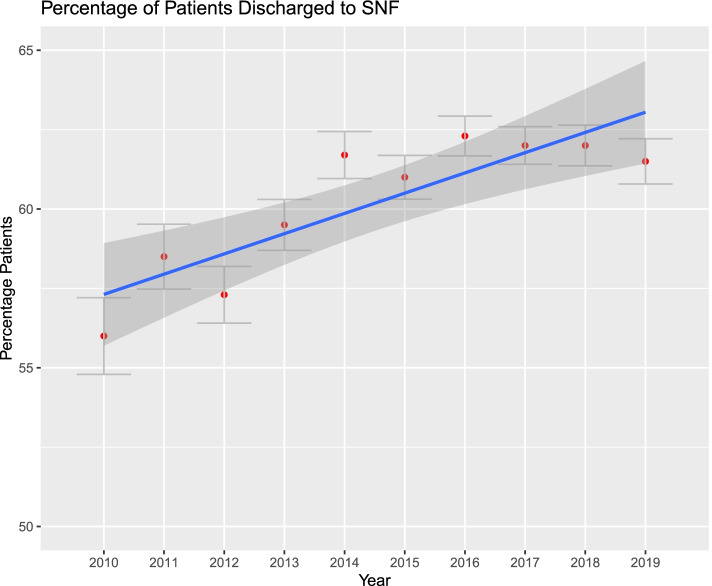
Percentage of patients discharged to a skilled nursing facility, from 2010 to 2019. The percentage patients increased from 56.0% (95%CI: 54.8%-57.2%) to 61.5% (95%CI: 60.8%-62.2%)

**Fig. 5:**
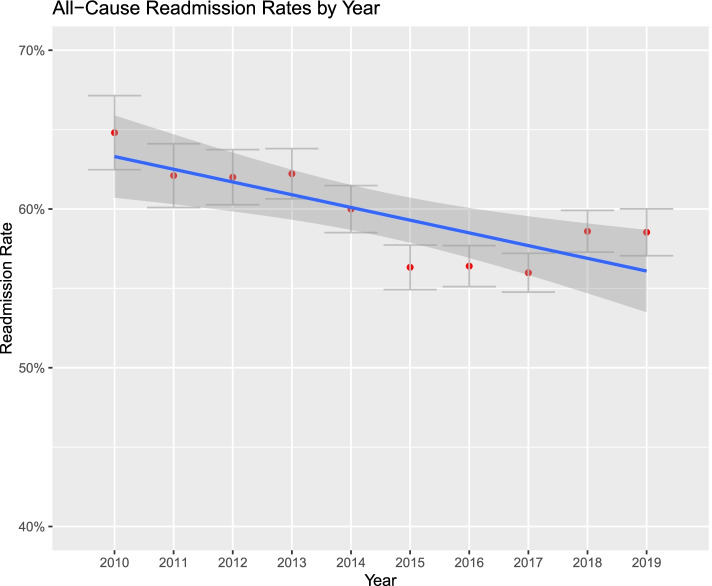
Percentage of patients readmitted for any cause, within 12 months of index, from 2010 to 2019. The percentage readmission decreased from 64.8% (95%CI: 62.5%-67.1%) to 58.5% (95%CI: 57.0%-60.0%)

**Fig. 6:**
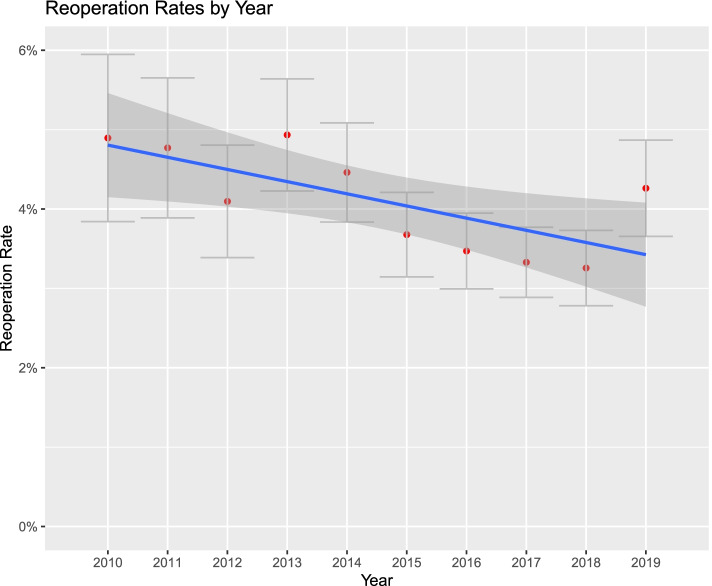
Percentage of patients with a hip reoperation within 12 months, from 2010 to 2019. The percentage reoperation decreased (non-significantly) from 4.89% (95%CI: 3.84%-5.96%) to 4.26% (95%CI: 3.65%-4.86%)

Figures 1, 2, 3, 4, 5 and 6. From 2010 to 2019, patient comorbidities increased significantly from 3.0 (95%CI: 2.9–3.1) to 3.4 (95%CI: 3.3–3.5). The percentage patients treated within 48 h of admission also increased significantly, from 75.2% (95%CI: 74.3%-76.1%) to 84.3% (95%CI: 83.9%-84.6%). The LOS, however, decreased from 6.2 (95%CI: 6.0–6.4) to 5.6 (95%CI: 5.5–5.7) days, and SNF discharge also increased, from 56.0% (95%CI: 54.8%-57.2%) to 61.5% (95%CI: 60.8%-62.2%). Readmissions rates decreased from 64.8% (95%CI: 62.5%-67.1%) to 58.5% (95%CI: 57.0%-60.0%) but there was no difference in reoperation rates.

## Discussion

Our study was designed to understand the characteristics of patients treated with intramedullary nails, for proximal femur fracture. Recent studies have demonstrated that TFNA nails had similar revision and breakage risks, compared to other devices [8, 9]. Our study was thus design to further understand the patient population, overall healthcare utilization and readmission rates, in patients treated with TFNA vs comparators. Overall, patients in our study had many comorbidities and a high risk for mortality. This finding comes as no surprise to practicing surgeons, however, to the best of our knowledge, the extent of the severity of these patients has not been documented in a large population analysis. We also observed that more than half of all patients would be readmitted for any cause within a year of index surgery. This finding also highlights the severity of these patients and their significant healthcare needs. Improving surgery outcomes by addressing underlying comorbidities has been discussed in other papers [14]. Inoue et al. provided some strategies to improve outcomes, some as basic as treating nutritional problems and evaluating possible sarcopenia in this population [14, 15].

The outcomes we analyzed (OR time, length of stay, discharge disposition, readmission and reoperation) were fairly consistent across all devices used. Reoperation was uncommon, at approximately 4%. This finding is in line with other papers that reported approximately 5% reoperations after IM nail use [16]. Goodnough et al. reported a 1.8–1.9% revision rate, but the definition of revision in this paper was limited to operations “in which a component was removed and/or replaced” [9]. Our definition was broader and included any reoperation of the hip. Our analysis also showed that reoperation rates also stayed fairly constant, with non-significant downward trends, between 2010 and 2019.

Our study has some key strengths: we used 10 years of data and were able to identify more than 40,000 patients, thus generating one of the largest intramedullary nail studies to date. The data we used has very detailed information on the care and hospital episode.

Interestingly, we also analyzed length of stay and Elixhauser Index as a function of time. A steady decline in duration of hospitalization has been observed, despite increase in patient comorbidity. This finding may be aligned with current hospital protocols to accelerate recovery and mobility post-surgery, along with the focus on operating on patients early on (within 48 h) of admission. The shortened length of stay did not affect reoperations, which did not change significantly during that time frame.

Our study has also some significant limitations: Patients treated within a hospital contributing data to PHD for index, but who change hospitals within 12 months and get readmitted or reoperated elsewhere would not show up as readmission or reoperation in our analysis. Similarly, patient outpatient or nursing home/hospice care is not captured in PHD. As is always the case with database research, we depend on accurate coding for diagnostic and procedures, and could not independently verify diagnoses or procedures. However, it is worth mentioning that PHD does significant data validation and has been used for more than 600 peer-reviewed publications. An additional limitation of our work is that we did not try and match patients across device types to compare them using comparative effectiveness methods. As such, data for TFNA and the non-TFNA cohort are shown separately in a descriptive method.

## Conclusions

Patients requiring intramedullary nail fixation for treatment of proximal femoral fractures are at high risk for severe conditions and mortality. Readmission was very common, affecting 60% of patients. The reoperation rates, however, were much lower, at approximately 4%. These findings were consistent in the TFNA and the non-TFNA cohorts. The 10-year analysis further highlighted increased patient comorbid conditions with time, but shorter length of stay and greater post-operative reliance on SNF.

## Data Availability

The data for these analyses were made available to the authors by third-party licenses from PREMIER (https://products.premierinc.com/applied-sciences/solutions/applied-research-and-analytics), a data provider in the US. Under the licensing agreement, the authors cannot provide raw data themselves. Other researchers could access the data by purchase through PREMIER, and the inclusion criteria specified in the Methods section would allow them to identify the same cohort of patients we used for these analyses.

## Abbreviations

ACR: All-cause readmission
AIS: Abbreviated Injury Scale
APR: All-patient refined
CI: Confidence interval
EI: Elixhauser Index
GLM: Generalized linear model
HCU: Healthcare Utilization
IRB: Institutional Review Board
ISS: Injury Severity Score
LOS: Length of hospital stay
ORT: Operating room time
PHD: Premier Healthcare Database
SD: Standard Deviation
SNF: Skilled Nursing Facility
TFN: Trochanteric Fixation Nail
TFNA: Trochanteric Fixation Nail Advanced
